# Draft genome and antibiotic resistance profile of a multidrug-resistant *K*. *pneumoniae*-ST152 strain KA017_S253 from a neonatal blood culture in Ghana

**DOI:** 10.1128/mra.00588-25

**Published:** 2025-08-11

**Authors:** Francis Kwame Morgan Tetteh, Beverly Egyir, William Boateng, Christian Owusu-Nyantakyi, Justice Kwesi Danso, Kashif Haq, Eric Sampane-Donkor, Kwabena Obeng Duedu

**Affiliations:** 1 Department of Medical Microbiology, University of Ghana Medical School 63533, Accra, Ghana; 2 Pathology Division, 37 Military Hospital, Accra, Ghana; 3 Department of Life and Sport Sciences, Birmingham City University 1725, Birmingham, United Kingdom; 4 Department of Bacteriology, Noguchi Memorial Institute for Medical Research, College of Health Sciences, University of Ghana 58835, Accra, Ghana; Wellesley College, Wellesley, Massachusetts, USA

**Keywords:** *Klebsiella pneumoniae*, sepsis, Ghana, antibiotic resistance

## Abstract

Bloodstream infection and neonatal sepsis pose a significant public health threat in sub-Saharan Africa. To support drug discovery, surveillance, and vaccine development prospects, genomic data on resistance are needed. We present a draft genome and antibiogram of a multidrug-resistant *Klebsiella pneumoniae-*ST152 strain KA017_S253, recovered from a neonate.

## ANNOUNCEMENT

*Klebsiella pneumoniae* is a common cause of gram-negative bacteremia and the leading cause of neonatal sepsis cases in sub-Saharan Africa (1, 2). The emergence of multidrug-resistant strains is on the rise, contributing to higher mortality rates (3). Whole-genome sequencing enables rapid identification and efficient antimicrobial resistance (AMR) surveillance (4). Here, we report the draft genome sequence and antibiogram of a multidrug-resistant *K. pneumoniae*-ST152 strain KA017_S253 recovered from the blood culture of a 7-day neonate with sepsis during a cross-sectional study in southern Ghana between April and December 2021.

The top of frozen skimmed-milk tryptone glucose glycerol stock of the *K. pneumoniae* was scraped with a sterile pipette tip and inoculated into tryptic soy broth (Oxoid, UK), incubated at 37°C for 24 h, after which a loopful was plated onto nutrient agar (Oxoid, UK) and incubated for 20 h at 37°C. The species of the isolate was confirmed using BD MALDI-TOF version 4.2.100 analyzer (Bruker Daltonics, Germany). Antibiotic susceptibility (AST) was performed using the minimum inhibition concentration (MIC) method on the MicroScan autoSCAN-4 system (American MicroScan, USA) according to the manufacturer’s instructions and interpreted using the CLSI M100 guidelines (5). Briefly, the isolate was inoculated into the microdilution panel containing preloaded antibiotics. The inoculated plate was then incubated for 20 h at 37°C and scanned for absorbance representing growth. *K. pneumoniae* NCTC 13438 and *Escherichia coli* ATCC 25922 were used as the quality control strains for the MIC test (Supplementary Data https://zenodo.org/records/15881304). The autoSCAN-4 performs automated AST in a 96-well microbroth dilution plate and is read by measuring the amount of light passing through the wells (6). The study was approved by the University of Ghana College of Health Sciences Ethics and Protocol Review Committee (ID:CHS-Et/M.2-P4.6/2021-2022).

Genomic DNA was extracted from an overnight culture of the isolate grown in nutrient broth using the QIAamp DNA minikit (Qiagen GmbH, Germany). Sequencing libraries were prepared with the DNA preparation (M) tagmentation kit (Cat: 20060059) (Illumina Inc., USA). The process involved DNA fragmentation, tagmentation, addition of index sequences, and amplification of indexed fragments. Libraries were diluted to a concentration of 2 nM, pooled, and sequenced on the NextSeq 1000 platform (Illumina Inc., San Diego, CA, USA) using 2 × 150 bp chemistry. DNA concentration and fragment sizes during the processes were assessed using the Qubit 1X dsDNA HS Assay read on the Qubit 4 Fluorometer (ThermoFisher Scientific, USA) and the 2100 Bioanalyzer system (Agilent Technologies, USA), respectively.

Indexes and reads with quality scores of <20 were trimmed using Trimmomatic v.0.39 (7), and quality control checks were performed with FastQC v.1.0 (8). Trimmed reads were assembled using Unicycler v.0.5.0 (9) and evaluated using QUAST v.5.2.0 (10). Sequence quality metrics were set at: Q-scores >30, minimum coverage of 20×, and minimum contig size of 200 bp. The assembled sequence data were analyzed using Kleborate v.3.1.3 (11) for resistance and virulence genes, multilocus sequence type, and serotype. The PlasmidFinder v.2.1 server (12) was used to detect plasmid replicons. The genome was annotated using the NCBI Prokaryotic Genome Annotation Pipeline v.6.10 (13). The results are summarized below (Table 1).

**TABLE 1:** Genomic profile of *K. pneumoniae^a^*

Acquired resistance genes (Kleborate prediction)	
Strain name: KA017_S253	
Family	Predicted genes
Aminoglycosides	aac(6′)-Ib-cr; aadA2; aph(3′)-VI; armA
Sulfonamides	sul1; sul2
Cephalosporins (3rd gen.)	blaCTX-M-15
Carbapenems	blaNDM-1; blaOXA-48
Tetracyclines	tet(A) (homolog)
Trimethoprim	dfrA12; dfrA14 (homolog)
Fluoroquinolones	qnrB1
Penicillins	OXA-1; SHV-1
Macrolides	mphE; msrE
Serotypes	K locus = KL149; O locus = O4
Multilocus sequence typing	152
Plasmids (Plasmid Finder)	IncFII(pBK30683); IncL; IncFIB(pNDM-Mar); IncHI1B(pNDM-MAR); ColRNAI; repB(R1701); Col440I
Genome size (bp)	5,590,943
N50 (bp)	351,825
GC content (%)	57
No. of reads	14,500,768
No. of contigs	107
Size of largest contig (bp)	621,991
No. of genes	5,584
No. of coding sequences	5,490
No. of rRNAs	7
No. of tRNAs	77
No. of ncRNAs	10
Coverage (×)	366
Genome accession no.	JBNYYN000000000
BioSample accession no.	SAMN48630582
SRA accession no.	SRR33648976
BioProject accession no.	PRJNA1265775

^*a*^ No virulence genes were determined for the organism using Kleborate.

## Data Availability

Assembled data were deposited in the National Center for Biotechnology Information (NCBI) database under the BioProject accession number PRJNA1265775 (GCA_050572995.1). This whole-genome shotgun project was deposited in GenBank under the accession number JBNYYN000000000. The raw read was deposited in the SRA under accession number SRR33648976 (https://trace.ncbi.nlm.nih.gov/Traces/?view=run_browser&acc=SRR33648976&display=metadata).

## References

[1] Verani JR, Blau DM, Gurley ES, Akelo V, Assefa N, Baillie V, Bassat Q, Berhane M, Bunn J, Cossa ACA, et al.. 2024. Child deaths caused by Klebsiella pneumoniae in sub-Saharan Africa and south Asia: a secondary analysis of Child Health and Mortality Prevention Surveillance (CHAMPS) data. Lancet Microbe 5:e131–e141. doi:10.1016/S2666-5247(23)00290-2 38218193 PMC10849973

[2] Okomo U, Akpalu ENK, Le Doare K, Roca A, Cousens S, Jarde A, Sharland M, Kampmann B, Lawn JE. 2019. Aetiology of invasive bacterial infection and antimicrobial resistance in neonates in sub-Saharan Africa: a systematic review and meta-analysis in line with the STROBE-NI reporting guidelines. Lancet Infect Dis 19:1219–1234. doi:10.1016/S1473-3099(19)30414-1 31522858

[3] Abdelsalam NA, ElBanna SA, Mouftah SF, Cobo-Díaz JF, Shata AH, Shawky SM, Atteya R, Elhadidy M. 2024. Genomic dynamics of high-risk carbapenem-resistant Klebsiella pneumoniae clones carrying hypervirulence determinants in Egyptian clinical settings. BMC Infect Dis 24:1193. doi:10.1186/s12879-024-10056-1 39438795 PMC11515790

[4] Köser CU, Ellington MJ, Peacock SJ. 2014. Whole-genome sequencing to control antimicrobial resistance. Trends Genet 30:401–407. doi:10.1016/j.tig.2014.07.003 25096945 PMC4156311

[5] Lewis II JS, Mathers A, Bobenchik AM, Bryson AL, Campeau S, Cullen SK, Dingle T, Romney M, Esparza G, Romney M. Humphries, Kirn TJJ, Lutgring J, et al.. 2025. Performance standards for antimicrobial susceptibility testing. In CLSI M100. Clinical and Laboratory Standards Institute, Malvern, PA, United States.

[6] Baker CN, Stocker SA, Rhoden DL, Thornsberry C. 1986. Evaluation of the MicroScan antimicrobial susceptibility system with the autoSCAN-4 automated reader. J Clin Microbiol 23:143–148. doi:10.1128/jcm.23.1.143-148.1986 3700598 PMC268589

[7] Bolger AM, Lohse M, Usadel B. 2014. Trimmomatic: a flexible trimmer for Illumina sequence data. Bioinformatics 30:2114–2120. doi:10.1093/bioinformatics/btu170 24695404 PMC4103590

[8] S. A. 2010. FastQC: a quality control tool for high throughput sequence data

[9] Wick RR, Judd LM, Gorrie CL, Holt KE. 2017. Unicycler: Resolving bacterial genome assemblies from short and long sequencing reads. PLoS Comput Biol 13:e1005595. doi:10.1371/journal.pcbi.1005595 28594827 PMC5481147

[10] Gurevich A, Saveliev V, Vyahhi N, Tesler G. 2013. QUAST: quality assessment tool for genome assemblies. Bioinformatics 29:1072–1075. doi:10.1093/bioinformatics/btt086 23422339 PMC3624806

[11] Lam MMC, Wick RR, Watts SC, Cerdeira LT, Wyres KL, Holt KE. 2021. A genomic surveillance framework and genotyping tool for Klebsiella pneumoniae and its related species complex. Nat Commun 12:4188. doi:10.1038/s41467-021-24448-3 34234121 PMC8263825

[12] Carattoli A, Zankari E, García-Fernández A, Voldby Larsen M, Lund O, Villa L, Møller Aarestrup F, Hasman H. 2014. In silico detection and typing of plasmids using plasmidFinder and plasmid multilocus sequence typing . Antimicrob Agents Chemother 58:3895–3903. doi:10.1128/AAC.02412-14 24777092 PMC4068535

[13] Tatusova T, DiCuccio M, Badretdin A, Chetvernin V, Nawrocki EP, Zaslavsky L, Lomsadze A, Pruitt KD, Borodovsky M, Ostell J. 2016. NCBI prokaryotic genome annotation pipeline. Nucleic Acids Res 44:6614–6624. doi:10.1093/nar/gkw569 27342282 PMC5001611

